# Physical education grades as markers of hedonic motivation to move: affective experiences from PE in adolescent girls and boys

**DOI:** 10.3389/fspor.2026.1922786

**Published:** 2026-10-14

**Authors:** Jona Kunz, Ralf Brand

**Affiliations:** Department of Sport and Health Sciences, Sport and Exercise Psychology, University of Potsdam, Potsdam, Germany

**Keywords:** adolescence, affect, exercise motivation, grading, school physical education

## Abstract

**Background:**

Physical education (PE) grades represent one educational indicator routinely available within many school systems. Because PE unfolds in a highly visible and embodied context, grades may also reflect affective-motivational processes. Understanding the information they contain is therefore practically relevant. This study explored whether PE grades are associated with students' hedonic motivation (antipathy–attraction) toward PE, sport, and exercise, and whether these associations differ between girls and boys.

**Methods:**

A total of 547 German school students (297 girls, 250 boys; 10–19 years old) completed a PE-specific version of the Affective Exercise Experiences Questionnaire (AFFEXX-PEG) and reported their two most recent PE grades. A linear mixed-effects model examined associations between grades and antipathy–attraction.

**Results:**

Better PE grades were associated with higher antipathy–attraction scores among both girls [*b* = 0.38, 95% CI (0.33, 0.44)] and boys [*b* = 0.26, 95% CI (0.19, 0.32)]. Boys reported higher antipathy–attraction scores than girls [*b* = 0.84, 95% CI (0.65, 1.03)]. The association between grades and antipathy–attraction was stronger among girls than among boys [*b* = −0.13, 95% CI (−0.21, −0.04)]. Boys tended to report positive antipathy–attraction scores even with modest PE grades, whereas among girls, predicted motivation typically shifted into the positive range only at better grades.

**Conclusion:**

The findings suggest that PE grades may function not only as indicators of students' standing in PE but also as markers of their affective motivation toward PE, sport, and exercise. Used reflectively, grades may therefore provide a prompt for teachers to explore and address this motivational dimension more directly, particularly among girls.

## 1 Introduction

Worldwide, students' school performance is commonly evaluated through grades (1). This also applies to PE, where assessment and grading represent central components of school practice (2). Because grades are routinely available for virtually all students, understanding what information they contain is of considerable educational relevance. While grades are typically interpreted as indicators of achievement, they may also reflect broader behavioral, motivational, and affective processes that unfold within educational settings.

This possibility may be particularly evident in PE. Unlike most academic subjects, PE evaluates students in a highly visible, embodied, and socially comparative context. However, the specific aims, curricula, and assessment practices of PE differ across educational systems. For example, in Germany and several other European contexts, PE is shaped by educational aims that extend beyond performance alone and include movement-related, social, cognitive, and health-oriented development (3, 4). Within these PE settings, students are assessed not only on what they know, but also on how they move, participate, perform, and present themselves in front of others (5–7). Accordingly, PE assessment in Europe is described as complex and partly controversial because grading practices often extend beyond isolated performance outcomes and may include dimensions such as effort, participation, engagement, behavior, and teachers' personal or internalized criteria (8–11).

This multidimensional understanding is also reflected in German sport-pedagogical approaches to assessment, which emphasize that PE grades should incorporate not only sport-motor performance but also individual progress and effort, social participation, and cognitive achievement (12). Qualitative research with German PE teachers suggests that these dimensions are also reflected in grading practice: although sport-motor performance generally receives the greatest weight, teachers additionally consider progress, effort, and social and cognitive competencies, with their relative weighting varying across educational stages and grading contexts (13).

This broader perspective on assessment is relevant because PE is commonly expected not only to develop movement-related competencies but also to foster positive attitudes toward physical activity (PA) and lifelong engagement in movement-related behaviors (14). This broader educational aim is particularly important given the globally low levels of PA among children and adolescents, especially among girls, who consistently report lower activity levels than boys across age groups (15, 16). Against this background, a more specific question concerns which affective-motivational characteristics may be reflected in PE grades and, consequently, what information these routinely assigned evaluations may contain beyond achievement alone.

One psychological perspective that may clarify this link concerns the role of affective experiences in shaping enduring motivation for PA and exercise. Hedonic motivation, the tendency to approach experiences that feel pleasant and avoid those that feel unpleasant, is considered a fundamental driver of human behavior (17, 18). From a neuroscience perspective, feelings of pleasure and displeasure are closely linked to valuation processes that guide learning and influence future behavioral choices (19). Applied to the domain of PA, this principle suggests that individuals are more likely to repeat movement experiences associated with pleasant feelings and avoid those linked to unpleasant feeling states. Accordingly, contemporary exercise psychology increasingly emphasizes affective responses during PA and exercise as central to understanding both immediate and longer-term engagement in movement behavior (20–23).

A key theoretical framework for understanding how affective experiences shape future movement behavior is the Affective-Reflective Theory of Physical Inactivity and Exercise (ART; 24). ART focuses on the psychological processes that occur when individuals encounter exercise-related opportunities. According to the theory, repeated pleasant and unpleasant experiences during exercise contribute to the formation of automatic affective valuations toward such stimuli. Through associative learning, these affective valuations gradually translate into automatically activated behavioral impulses toward future opportunities. Pleasant movement experiences are assumed to strengthen an approach impulse, whereas unpleasant experiences may reinforce an avoidance impulse. When individuals subsequently encounter opportunities for PA, these impulses are assumed to become activated and may influence subsequent movement-related decisions and behavior.

Building on this perspective, the Affective Exercise Experiences framework (AFFEXX; 25) conceptualizes affective exercise experiences as a summative representation of one's exercise-related affective history. These experiences differ in their hedonic tone and collectively shape how people perceive and relate to exercise. Within this framework, antipathy–attraction toward exercise captures the extent to which individuals are affectively inclined to seek out or avoid (i.e., hedonic wanting; 19) movement experiences. Attraction reflects a tendency to approach and engage in movement contexts, whereas antipathy reflects a tendency toward avoidance and disengagement.

Empirical evidence supports the relevance of the AFFEXX framework for understanding PA behavior. Although the AFFEXX questionnaire has also been used with adolescents, the strongest empirical support for its central propositions currently comes from adult samples. For example, Brand et al. (26) showed that affective exercise experiences are meaningfully related to PA, with pleasant core affective experiences predicting exercise behavior particularly when accompanied by strong attraction toward exercise. PE-specific studies similarly suggest that constructs such as enjoyment and autonomous motivation are associated with students' persistence, motivational orientations, and PA-related outcomes both within and beyond PE (27–30). Taken together, these findings highlight the importance of affective-motivational characteristics in understanding students' engagement in PA contexts.

Given its near-universal reach among children and adolescents, PE provides a particularly relevant context for the development of lasting movement-related orientations. Consistent with this assumption, motivational processes in PE have been associated with PA participation beyond school settings (30, 31).

Because PE unfolds within a formal evaluative context, students' affective-motivational orientation toward participation may become visible through behavioral expressions such as effort, engagement, persistence, reluctance, or withdrawal during lessons. Many of these behaviors overlap with dimensions that have repeatedly been discussed in relation to PE assessment and grading practices (6–8, 32). Consequently, it is conceivable that students’ affective exercise experiences and their resulting attraction or antipathy toward participation may be indirectly reflected in PE grades.

Importantly, this reasoning does not imply that PE grades directly assess affective experiences or motivation, nor does it resolve the direction of any potential association. Rather, it motivates the exploratory question of whether routinely assigned PE grades systematically covary with students' hedonic orientation toward participation.

This perspective is consistent with broader educational research linking emotions, motivation, engagement, and achievement (33, 34). Within PE, motivational orientations, effort, engagement, and achievement-related outcomes have repeatedly been shown to covary (35–41).

An additional reason why these questions may be worth exploring concerns recurring sex differences in both PE and participation in sport and exercise more broadly. Whereas girls tend to outperform boys and receive higher grades across many academic domains, including languages and mathematics (42), boys often receive better grades in PE across different national contexts (43–47). At the same time, girls consistently report lower levels of PA than boys (15, 16). Moreover, studies examining conceptually related constructs, such as enjoyment and autonomous motivation in PE, have often reported sex-specific patterns, with girls tending to show less favorable profiles than boys (27–29).

From a psychological perspective, the coexistence of these patterns raises an intriguing possibility. If affective exercise experiences contribute to the development of attraction or antipathy toward participation, and if such tendencies become visible through students' behavior during PE, then affective-motivational processes may represent one factor (among several involved) in the sex-specific patterns repeatedly observed in PE. Although existing research cannot establish such connections conclusively, this possibility warrants empirical attention.

Despite extensive research on PE motivation, engagement, and achievement, little is known about whether PE grades are systematically associated with students' affective-motivational characteristics. Contemporary exercise psychology provides theoretical reasons why such associations might exist, whereas educational research has extensively examined PE grading. Surprisingly, however, these two perspectives have rarely been linked directly. Before questions concerning causal direction, explanatory mechanisms, or practical implications can be addressed, it first seems necessary to establish whether these two conceptually distinct domains are empirically related at all.

The present study therefore adopted an explicitly exploratory perspective. Rather than testing explanatory mechanisms or causal pathways, we examined whether students' PE grades are systematically associated with their hedonic motivation toward PE and exercise as assessed with a PE-specific German version of the AFFEXX questionnaire (25). In doing so, we sought to provide an initial empirical link between educational assessment and contemporary exercise psychology.

Because previous research has consistently reported sex differences in PE grades, affective experiences, and physical activity participation, we further explored whether this association differed between girls and boys. Thus, the study addressed two straightforward questions: whether PE grades are associated with students' hedonic motivation toward PE and exercise, and whether this association differs between girls and boys.

## 2 Materials and methods

### 2.1 Participants

A total of 547 students from Germany participated in the study (297 identified as female and 250 as male). Participation was voluntary and required written informed consent from a parent or legal guardian as well as written informed assent from the participating student. The study protocol, including the recruitment procedure, consent materials, and survey instruments, was reviewed and approved by the ethics committee of the University of Potsdam (approval number: 102/2025).

Participants were enrolled in grades 5 through 13 and ranged in age from 10 to 19 years (*M* = 14.6, *SD* = 1.8). Based on grade level, the sample comprised 123 students in lower-secondary school (grade levels 5–7; *M* = 12.4 years, *SD* = 0.9; 56% female), 343 in middle-secondary school (grade levels 8–10; *M* = 14.8 years, *SD* = 1.1; 56% female), and 81 in upper-secondary school (grade levels 11 and above; *M* = 17.1 years, *SD* = 1.6; 45% female). School-type information was available for 543 participants: 239 students (44.0%) attended a Gymnasium, 186 (34.3%) an integrated secondary school (ISS), 81 (14.9%) a comprehensive school (Gesamtschule), 20 (3.7%) a primary school (Grundschule), 10 (1.8%) another school type, and 7 (1.3%) a community school.

At the time of data collection, 57.5% of students reported being active members of a sports club. In Germany, school PE is mandatory, and club sports represents one of the most common forms of organized PA beyond school. National surveys report club membership rates of 60%–65% among adolescents (48), suggesting that activity levels in our sample are broadly in line with national trends.

No formal *a priori* power analysis was conducted. The final analytic sample comprised 547 students. Given the exploratory nature of the study, we therefore focus on effect estimates and their corresponding 95% confidence intervals rather than interpreting statistical significance in relation to a prespecified power target. At the same time, the sample size is well within the range commonly encountered in quantitative research involving student populations (49), supporting reasonably precise estimation of the reported associations.

### 2.2 Measures

#### 2.2.1 Hedonic motivation (AFFEXX-PEG)

To assess students' hedonic motivational tendencies toward PE and exercise, we used the AFFEXX Physical Education German version (AFFEXX-PEG), a PE-specific version of the German AFFEXX questionnaire (AFFEXX-G; 26), which in turn is based on the original AFFEXX instrument (25). Item wording was adapted to ensure age-appropriate language and contextual relevance for adolescents in PE settings. Preliminary piloting with students from the target age group supported item clarity and comprehensibility.

Like other versions of the AFFEXX questionnaire, the AFFEXX-PEG consists of 36 bipolar items rated on a seven-point scale ranging from −3 (very negative) to +3 (very positive). Psychometric analyses conducted prior to the main analyses supported the reliability and factorial validity of all AFFEXX-PEG subscales. For the purposes of the present study, analyses focused exclusively on the hedonic motivation subscale “antipathy–attraction”, which captures students' affectively grounded tendency to approach or avoid participation in PE. The subscale demonstrated good internal consistency in the present sample (Cronbach's *α* = 0.86), with good internal consistency among girls (*α* = 0.85) and acceptable among boys (*α* = 0.78). Given the study's focus on sex differences, we additionally examined measurement invariance of the antipathy–attraction scale across girls and boys using multi-group confirmatory factor analysis. Full scalar invariance was not supported, but metric and partial scalar invariance were achieved, indicating that the scale functioned largely comparably across groups.

Detailed psychometric results, the anonymized dataset, analysis scripts, complete questionnaire materials, and exemplary English item translations are available in the project repository; see the Data Availability Statement at the end of the manuscript.

#### 2.2.2 PE-grades

Participants were asked to report the PE grades they had received on their last two school report cards (i.e., mid-year or end-of-year reports). These grades were averaged to calculate each student's mean PE grade. Because German secondary schools commonly use two different grading systems, depending on educational stage, reported grades originated from either a six-point scale (lower- and middle-secondary levels; 1 = very good, 6 = insufficient), or a 15-point scale (upper-secondary level; 15 = highest achievement, 0 = lowest achievement). To facilitate comparisons across students, all grades were converted to the 15-point scale for analysis (see Table 1 for an overview and comparison of the German, U.S., and U.K. grading systems).

**Table 1:** Comparison of German secondary school grades with U.K. and U.S.

Germany (GP/letter grade)	U.K. (GCSE)	U.S. (GPA/letter grade)	Description
15 (1+)	9		
14 (1)	8	4.0 (A)	Very good
13 (1−)	7		
12 (2+)			
11 (2)	6	3.0 (B)	Good
10 (2−)			
9 (3+)	5		
8 (3)		2.0 (C)	Satisfactory
7 (3−)	4		
6 (4+)	3		
5 (4)	2	1.0 (D)	Sufficient
4 (4−)	1		
3 (5+)			
2 (5)	U	0.0 (F)	Failed
1 (5−)			
0 (6)			

U.K. GCSE equivalents are based on Ofqual (50). U.S. GPA equivalents are based on the National Centre for Education Statistics (51). These grading systems represent broad national reference points and do not constitute official conversions; grading practices may vary by institution. GCSE, General Certificate of Secondary Education; GPA, grade point average.

### 2.3 Survey procedure

Students were recruited via their PE teachers using convenience-based access. Depending on local recruitment opportunities, participation comprised entire classes as well as smaller numbers of students from individual classes or schools. Participants completed the online questionnaire individually and privately, at school under teacher supervision. The survey was administered using the SoSci Survey platform (52) and hosted on a secure server licensed by the University of Potsdam.

Following a brief introduction, the survey began with demographic questions covering sex, age, school grade, school type, two most recent PE grades, and extracurricular sport engagement. The main section consisted of the 36 AFFEXX-PEG items, each presented on a separate screen to ensure focused responding. On average, participants completed the entire survey in 11:40 min (±03:00 min).

### 2.4 Data analysis

All statistical analyses were conducted using R version 4.6.0 (53) within the RStudio environment (54).

#### 2.4.1 Descriptive statistics

Descriptive statistics were calculated for all study variables. Sex differences in hedonic motivation and PE grades were examined using Welch two-sample *t*-tests. Effect sizes were calculated using Cohen's *d* with corresponding 95% confidence intervals.

#### 2.4.2 Main analysis

For descriptive purposes, a Pearson correlation was calculated to quantify the crude association between hedonic motivation and PE grades. The primary analysis extended this descriptive estimate by examining the adjusted association between hedonic motivation and PE grades while accounting for sex, the PE grade × sex interaction, and differences between school grade levels. Because school- and class-level identifiers were not consistently available across the convenience-based recruitment, clustering at these levels could not be modeled reliably. Grade level was included independently to account for potential developmental differences, as both PE grades and motivational experiences have been shown to vary across adolescence (35, 55). Accordingly, the primary analysis was conducted using a linear mixed-effects model including a random intercept for grade level. PE grades were mean centered prior to analysis to facilitate interpretation of the interaction model. To facilitate interpretation of the PE grade × sex interaction, sex-specific slopes were obtained from the fitted model, with girls as the reference group and the corresponding simple slope estimated for boys. Parameters were calculated using restricted maximum likelihood estimation, and approximate degrees of freedom and *p*-values were obtained using Satterthwaite's method. Model assumptions were inspected visually and indicated no substantial violations. Results are reported as unstandardized fixed-effect estimates (*b*) with standard errors, 95% confidence intervals, *t*-values, and *p*-values.

## 3 Results

### 3.1 Descriptive statistics and sex differences

Descriptive distributions and sex differences are illustrated in Figure 1. Girls reported significantly lower antipathy–attraction scores (*M* = 0.35; *SD* = 1.44) than boys (*M* = 1.47; *SD* = 1.11), *t*_(534.41)_ = −10.26, *p* < 0.001, *d* = −0.87, 95% CI [−1.04, −0.69]. Antipathy–attraction scores could range from −3, indicating strong antipathy, to +3, indicating strong attraction toward exercise with 0 representing a neutral midpoint. As illustrated in Figure 1A, girls' scores were distributed substantially closer to this neutral midpoint and extended further into the negative range compared to boys. Descriptively, 34% of girls reported antipathy–attraction scores below the neutral midpoint, compared to only 10% of boys.

**Figure 1:**
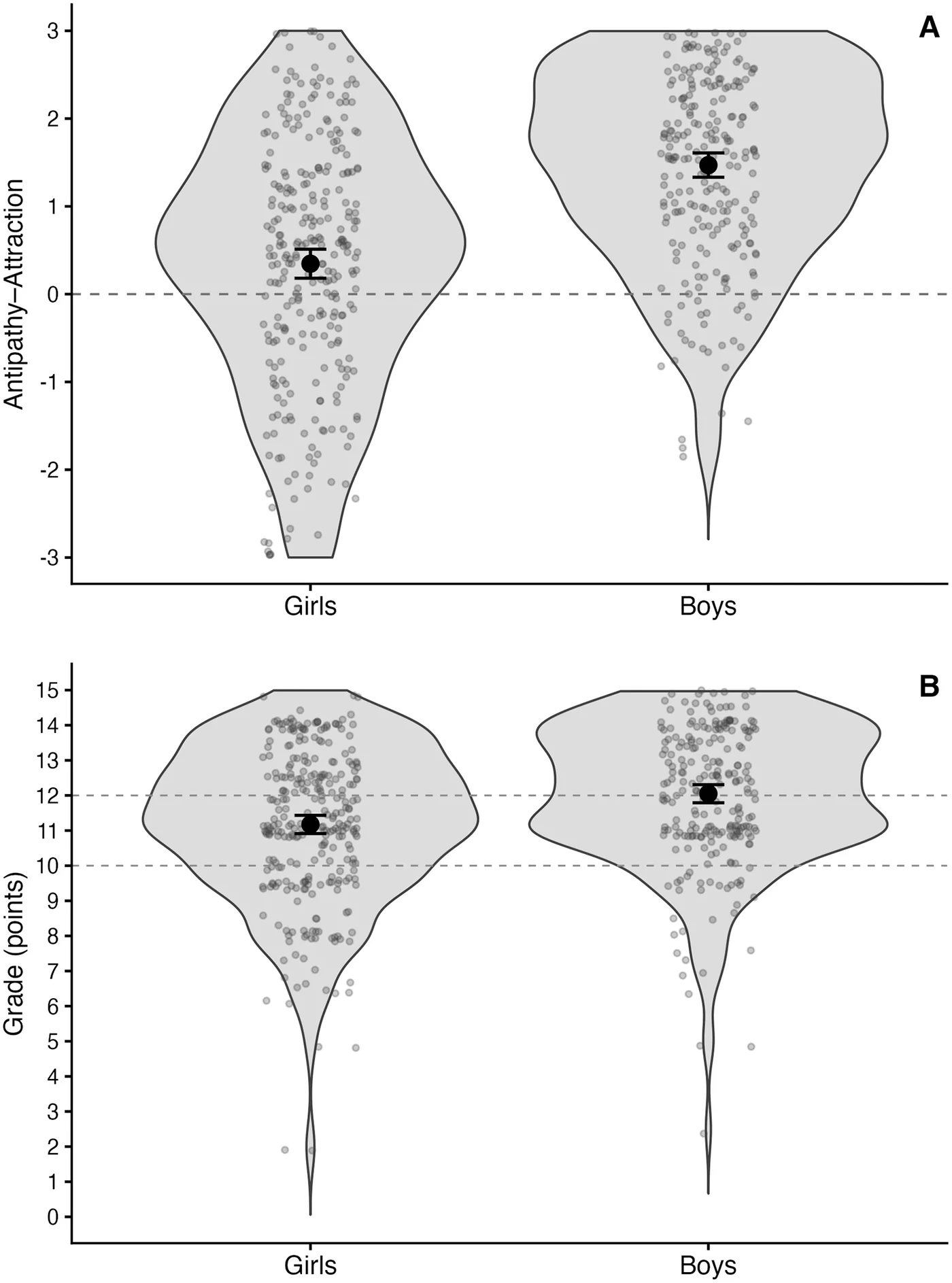
Distribution of hedonic motivation **(A)** and PE grades **(B)** by sex. Violin plots represent data distributions. Jittered points indicate individual observations. Black points represent group means, with error bars indicating 95% confidence intervals. In Panel **A**, the dashed horizontal line marks the neutral midpoint of the hedonic motivation scale. In Panel **B**, dashed horizontal lines indicate the range corresponding to the German “good” grade category.

Girls also showed significantly lower PE grades (*M* = 11.17; *SD* = 2.23) than boys (*M* = 12.10; *SD* = 2.00), *t*_(540.63)_ = −4.67, *p* < 0.001, *d* = −0.41, 95% CI [−0.58, −0.23]. As illustrated in Figure 1B, mean PE grades for both groups fell within the range corresponding to the German “good” grade category (roughly equivalent to a U.K. GCSE grade 6), although boys' grades tended somewhat more toward the “very good” range than girls' grades.

### 3.2 Main analysis

Hedonic motivation showed a substantial positive association with PE grades, *r* = 0.58, 95% CI [0.52, 0.63], *p* < 0.001, indicating that students with better PE grades generally reported more favorable affective-motivational orientations.

Results from the linear mixed model are presented in Table 2 and illustrated in Figure 2. The random-intercept variance was small, *τ*_00_ = 0.004, *SD* = 0.06, relative to the residual variance, *σ*^2^ = 1.147, *SD* = 1.07, ICC = 0.003, indicating minimal differences in hedonic motivation between school grade levels after accounting for PE grades, sex, and their interaction.

**Table 2:** Linear mixed model predicting hedonic motivation.

Predictor	*b*	***SE***	95% CI	***df***	*t*	*p*
Intercept	0.51	0.07	[0.37, 0.65]	6.16	7.19	<.001
PE grade	0.38	0.03	[0.33, 0.44]	521.23	13.52	<.001
Sex (boys)	0.84	0.10	[0.65, 1.03]	522.98	8.77	<.001
PE grade × Sex (boys)	−0.13	0.04	[−0.21, −0.04]	521.37	−2.90	.004

PE grades were mean-centered. Sex was coded with girls as the reference group. Grade level was included as a random intercept. Unstandardized fixed-effect estimates are reported. Degrees of freedom were estimated using Satterthwaite's method.

**Figure 2:**
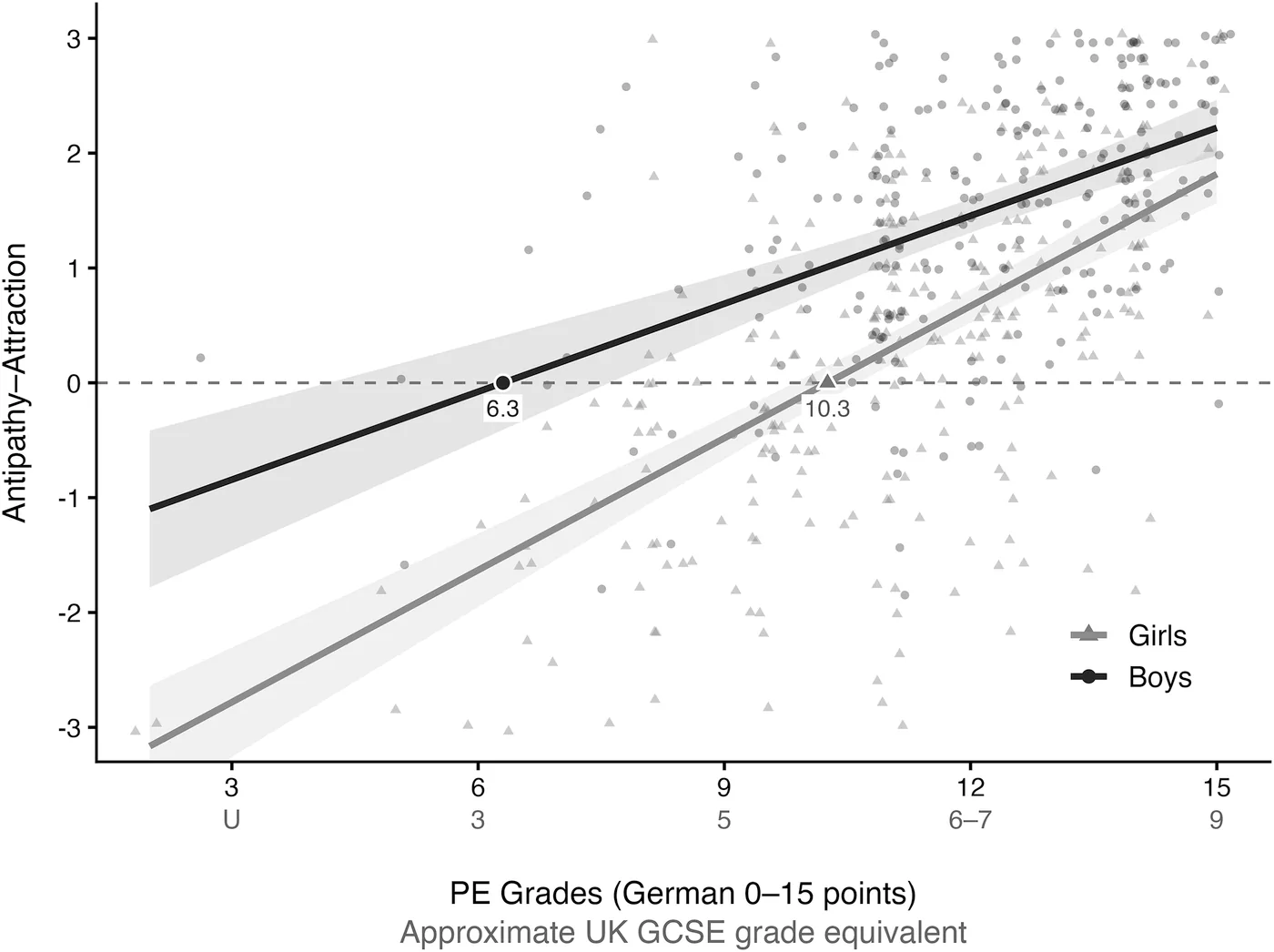
Association between PE grades and hedonic motivation by sex. Points represent individual observations; triangles indicate girls and circles indicate boys. Solid lines indicate model-predicted associations from the linear mixed-effects model, with 95% confidence intervals. The dashed horizontal line marks the neutral midpoint of the hedonic motivation scale, separating antipathy from attraction. The white-outlined markers on this line indicate the model-implied PE grade values at which predicted hedonic motivation reached the neutral midpoint for girls and boys, respectively. Approximate U.K. GCSE grade equivalents are shown below the x-axis.

In the linear mixed model, better PE grades were significantly associated with higher levels of hedonic motivation among girls, *b* = 0.38, *SE* = 0.03, *t*_(521.23)_ = 13.52, *p* < 0.001, 95% CI [0.33, 0.44], and among boys, *b* = 0.26, *SE* = 0.03, *t*_(498.15)_ = 7.55, *p* < 0.001, 95% CI [0.19, 0.32]. At the mean PE grade, boys reported significantly higher levels of hedonic motivation than girls, *b* = 0.84, *SE* = 0.10, *t*_(522.98)_ = 8.77, *p* < 0.001, 95% CI [0.65, 1.03].

In addition, the interaction between PE grades and sex was significant, *b* = −0.13, *SE* = 0.04, *t*_(521.37)_ = −2.90, *p* = 0.004, 95% CI [−0.21, −0.04], indicating that the positive association between PE grades and hedonic motivation was somewhat stronger among girls than boys.

As illustrated in Figure 2, model-implied crossover points suggested that predicted antipathy–attraction scores reached the neutral midpoint at substantially lower PE grades for boys than for girls. In other words, boys tended to report positive hedonic motivation even at comparatively modest grade levels, whereas among girls, predicted motivation typically shifted into the positive range only at higher PE grades.

## 4 Discussion

The present study explored whether routinely assigned PE grades are associated with students' hedonic motivational orientation toward PE and exercise. Three findings stand out. First, PE grades were strongly associated with hedonic motivation. Second, boys reported substantially more favorable hedonic motivational orientations than girls. Third, the association between PE grades and hedonic motivation differed by sex, with the relationship being stronger among girls than among boys.

Perhaps the most noteworthy finding is the strength of the association between PE grades and antipathy–attraction (*r* = 0.58). Although previous research has linked motivation, engagement, and achievement-related outcomes in PE (35–41), grades and antipathy–attraction toward movement represent conceptually distinct constructs. PE grades represent externally assigned evaluations of student performance and participation, whereas hedonic motivation captures students' affective orientation toward movement experiences. The observed association therefore suggests that PE grades may contain information extending beyond students' observable achievement and participation. Specifically, they may reflect aspects of students' affective-motivational relationship with movement that have received little attention in educational discussions of PE assessment. This observation provides an initial empirical bridge between educational assessment research and contemporary exercise psychology (6–8, 32).

Importantly, this finding should not be interpreted as indicating that PE grades directly measure motivation or affective experiences, nor do they permit conclusions about causal direction. Rather, grades and hedonic motivation may covary because both are expressed through students' behavior during PE. Students who approach PE with greater attraction may be more likely to engage more actively, persist when challenged, and invest greater effort, whereas students who are characterized by antipathy may participate more reluctantly or withdraw. Such behavioral tendencies are observable to teachers and may become reflected in grading practices. Conversely, repeated evaluative experiences associated with grading may themselves contribute to the development of students' affective orientations toward PE and exercise.

This interpretation is noteworthy because it points to a potentially useful convergence between educational practice and exercise psychology. Psychological assessments of affective-motivational orientations are rarely available in everyday school settings, whereas PE grades are routinely assigned to virtually all students. If future longitudinal research confirms the present findings, routinely available grades may provide educators with a pragmatic prompt to consider students whose affective-motivational experiences more closely.

This marker perspective may be particularly important for girls. Consistent with previous literature, girls reported less favorable motivational profiles than boys (27–29). In the present study, this difference was pronounced. Girls showed substantially lower hedonic motivation overall, which placed them closer to neutrality or antipathy even when their PE grades were still relatively favorable.

The grade-specific pattern made this difference particularly visible. Boys tended to remain on the attraction side of the scale across a broader range of PE grades, whereas girls reached the neutral point at comparatively better grades. This pattern was further sharpened by the significant interaction, indicating that the grade-motivation link was stronger among girls. Descriptively, 31.6% of girls had PE grades at or below the model-implied neutral point of 10.3 on the German grade-point scale, roughly equivalent to a U.K. GCSE grade 5–6. Among these girls, 70% reported antipathy. This is noteworthy because antipathy does not merely indicate reduced momentary enjoyment but can reflect an accumulated hedonic orientation against movement that is conceptually relevant to students' broader relationship with sport and PA beyond school. Thus, PE grades that appear satisfactory from an achievement perspective may nevertheless coincide with less favorable affective-motivational orientations, particularly among girls.

Viewed more broadly, the findings are compatible with the idea that educational evaluations and affective-motivational states in PE are more closely intertwined than typically assumed. Contemporary exercise psychology has repeatedly emphasized the importance of affective experiences for the development of enduring movement-related orientations (20–25, 56). Although the present study cannot address developmental mechanisms directly, the observed association raises the possibility that PE grades and students' affective-motivational orientations become linked over time through repeated experiences in PE. On the one hand, students' existing attraction or antipathy toward participation may shape how they engage, invest effort, or withdraw during lessons, thereby becoming visible in behaviors that may also contribute to grading. On the other hand, evaluative experiences in PE may themselves form part of the broader set of pleasant and unpleasant movement-related experiences through which students develop affective orientations toward exercise. ART provides a theoretical perspective on this latter process by proposing that repeated affective experiences associated with exercise-related situations contribute, through associative learning, to the formation of automatic affective valuations that can subsequently facilitate approach- or avoidance-oriented responses to movement opportunities (24, 56). In this sense, grading experiences may represent one element of the broader evaluative context in which such affective learning occurs. Seen from this perspective, the present findings do not explain how affective-motivational orientations develop, but they suggest that educational evaluations and exercise-psychological processes may be more closely intertwined than has generally been assumed. Exploring this intersection represents a promising direction for future research. Figure 3 illustrates one possible process through which it may unfold, together with potential individual and contextual moderators.

**Figure 3:**
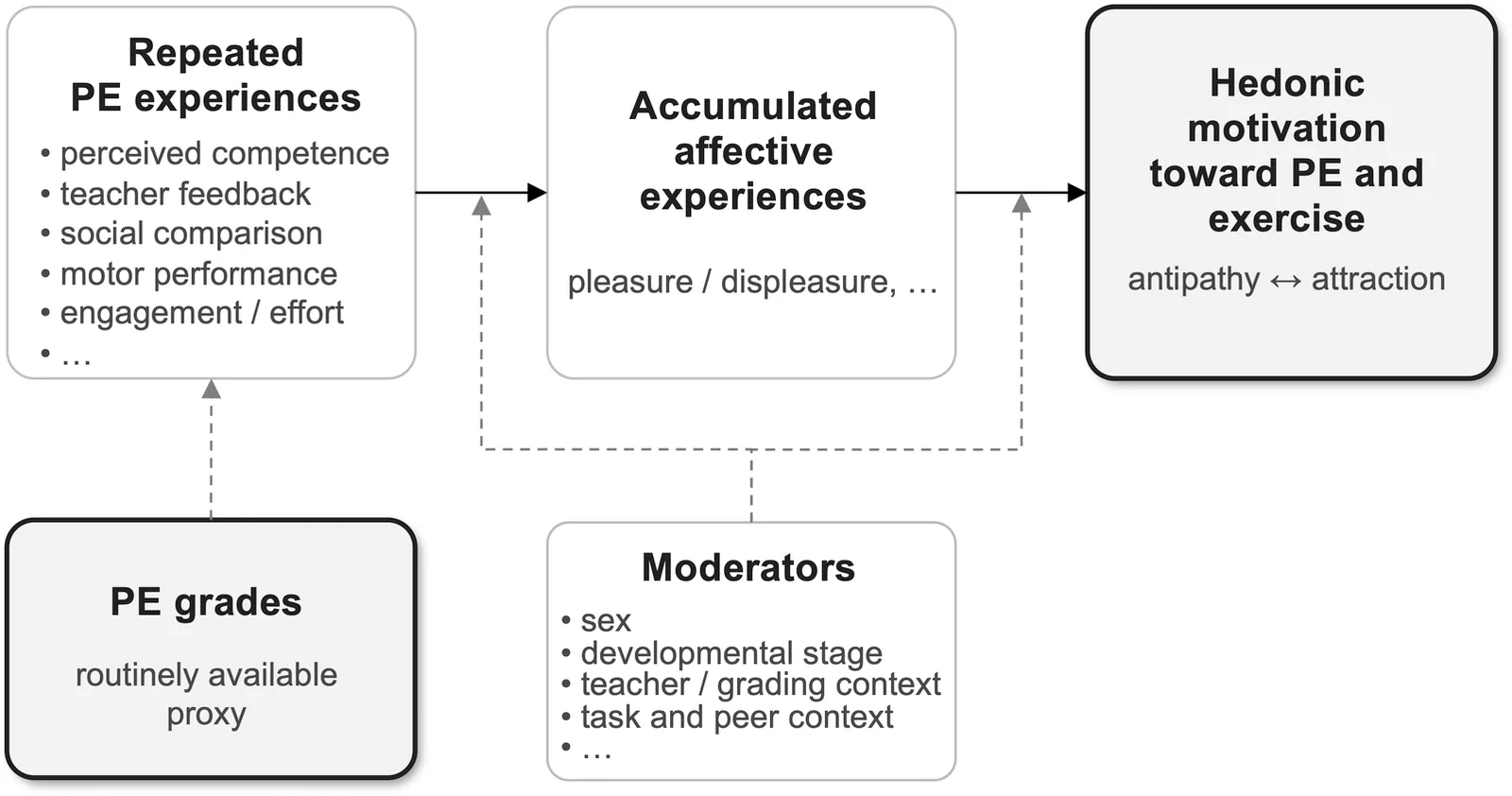
Conceptual process model linking repeated PE experiences, PE grades, and hedonic motivation. Solid arrows represent a proposed process linking repeated PE experiences, accumulated affective experiences, and hedonic motivation toward PE and exercise. PE grades are presented as a routinely available proxy that may reflect aspects of students’ experiences in PE, with the dashed arrow indicating this proposed indicator link. Dashed arrows from the moderator box indicate potential moderating influences. Although sex moderated the overall grade–motivation association, the proposed process and the moderating roles depicted in the model were not tested in the present study.

### 4.1 Limitations, strengths, and future directions

The main limitation of the present study is its cross-sectional design. Consequently, the findings describe associations and do not establish causal direction or the mechanisms underlying the observed relationship. It remains unclear whether affective-motivational orientations contribute to grade-relevant behaviors, whether grading experiences influence students' motivational development, or whether both processes unfold reciprocally over time. Longitudinal studies with repeated assessments are needed to establish temporal ordering and to examine these potential pathways, ideally incorporating relevant explanatory variables such as perceived competence, engagement, effort, and characteristics of the grading context, all of which have been implicated in students' motivational experiences and educational outcomes in PE (57–60).

A second limitation concerns the use and interpretation of self-reported PE grades. Although students reported the grades from their two most recent school report cards, recall inaccuracies as well as self-presentational or social-desirability effects cannot be ruled out. At a descriptive level, however, the grade distribution observed in the present sample appears broadly consistent with previous German and European data showing generally favorable PE grades and a tendency toward higher grades among boys (45, 46, 61). Importantly, this descriptive consistency does not verify the accuracy of individual self-reports. Future studies should therefore, where feasible, verify reported grades against official school records or report cards. Beyond the accuracy of individual reports, the use of overall report-card grades prevents us from determining whether the observed association with hedonic motivation was driven primarily by sport-motor performance or by other components of grading, such as progress, effort, or social and cognitive competencies.

Furthermore, the present findings should be interpreted considering the German sample and the broader European PE contexts in which educational aims commonly extend beyond performance alone to include movement-related, social, cognitive, and health-oriented development.

Despite these limitations, the study provides an informative first empirical step toward understanding the relationship between PE grades and affective-motivational processes. The sample was comparatively large, the observed associations were substantial, and the differences between boys and girls were broadly consistent with theoretical expectations and previous research.

## 5 Conclusion

This exploratory study, based on a sample of German adolescents, shows that PE grades are meaningfully associated with hedonic motivation toward PE and exercise, with a stronger association and less favorable motivational profiles among girls. At the same time, the sex-specific pattern cautions against interpreting a given PE grade as reflecting the same level of hedonic motivation for all students. When interpreted cautiously, grades may therefore offer teachers a pragmatic prompt to explore students' experiences more directly, for example through conversations or brief affective check-ins, particularly among girls. They should, however, complement rather than replace direct attention to students' affective-motivational experiences.

Longitudinal research using verified grades, repeated assessments, and more representative samples is needed to clarify how grading experiences, participation, and hedonic motivation become linked over time. By placing affective experience alongside achievement, this study points toward a broader understanding of successful PE: one concerned not only with how students perform, but also with the relationship with movement they develop.

## Data Availability

The datasets presented in this study can be found in online repositories. This data can be found here: https://doi.org/10.17605/OSF.IO/467B2.
